# Poly(3-hydroxybutyrate)/ZnO Bionanocomposites with Improved Mechanical, Barrier and Antibacterial Properties

**DOI:** 10.3390/ijms150610950

**Published:** 2014-06-17

**Authors:** Ana M. Díez-Pascual, Angel L. Díez-Vicente

**Affiliations:** 1 Institute of Polymer Science and Technology (ICTP-CSIC), Juan de la Cierva 3, Madrid 28006, Spain; 2 Airbus Operations S. L., John Lennon s/n, Getafe, Madrid 28906, Spain; E-Mail: aldiezvicente@gmx.es

**Keywords:** PHB, ZnO nanoparticles, antibacterial, barrier properties, food packaging

## Abstract

Poly(3-hydroxybutyrate) (PHB)-based bionanocomposites incorporating different contents of ZnO nanoparticles were prepared via solution casting technique. The nanoparticles were dispersed within the biopolymer without the need for surfactants or coupling agents. The morphology, thermal, mechanical, barrier, migration and antibacterial properties of the nanocomposites were investigated. The nanoparticles acted as nucleating agents, increasing the crystallization temperature and the degree of crystallinity of the matrix, and as mass transport barriers, hindering the diffusion of volatiles generated during the decomposition process, leading to higher thermal stability. The Young’s modulus, tensile and impact strength of the biopolymer were enhanced by up to 43%, 32% and 26%, respectively, due to the strong matrix-nanofiller interfacial adhesion attained via hydrogen bonding interactions, as revealed by the FT-IR spectra. Moreover, the nanocomposites exhibited reduced water uptake and superior gas and vapour barrier properties compared to neat PHB. They also showed antibacterial activity against both Gram-positive and Gram-negative bacteria, which was progressively improved upon increasing ZnO concentration. The migration levels of PHB/ZnO composites in both non-polar and polar simulants decreased with increasing nanoparticle content, and were well below the current legislative limits for food packaging materials. These biodegradable nanocomposites show great potential as an alternative to synthetic plastic packaging materials especially for use in food and beverage containers and disposable applications.

## 1. Introduction

The foremost role of food packaging is to preserve the quality and safety of food products during transportation and storage, and to extend their shelf-life by preventing unfavorable conditions such as spoilage microorganisms, chemical contaminants, oxygen, moisture, light, and so forth. The food package should hinder gain or loss of moisture, prevent microbial contamination, act as a barrier against permeation of water vapour, oxygen, carbon dioxide and other volatile compounds, have high mechanical strength, good thermal, chemical and dimensional stability, recyclability and biodegradability [1]. Over the past century, many synthetic polymers fabricated from non-renewable fossil fuels like polyethylene terephthalate (PET), polyvinylchloride (PVC), polyethylene (PE), polypropylene (PP), polystyrene (PS) and polyamide (PA) have been widely used as packaging materials due to their large availability at relatively low cost, good mechanical performance such as tensile and impact strength, good barrier properties, heat sealability, and so forth [2]. Nevertheless, the production of these synthetic polymers needs to be restricted since it leads to the exhaustion of petroleum resources. Moreover, these thermoplastics are not non-totally recyclable and/or biodegradable, and hence pose serious ecological problems like “white pollution” [3]; consequently, biodegradable alternatives are highly desired. In particular, poly(3-hydroxybutyrate) (PHB) is a fully biodegradable and biocompatible polyester synthesized by bacterial fermentation from renewable resources such as cane sugar that has recently attracted much attention as an alternative to petroleum-based polymeric materials [4]. PHB is an isotactic linear thermoplastic built of 3-hydroxy butyric acid with a chemical formula of [–O–CH–(CH_3_)–CH_2_–CO–]*_n_* that was first discovered in 1926 by Lemoigne and is now produced on an industrial scale. This biopolymer has already been applied in small disposable products and packaging materials [5]. However, it presents several shortcomings for commercial use such as high crystallinity (>50%), which leads to an inherent brittleness and poor impact resistance, a relatively high water vapour permeability (WVP) as well as low resistance to thermal degradation that causes the material to be easily degradable, which combined with a relatively high melting point (*ca.* 175 °C) results in a narrow processing window. To overcome these drawbacks with a view to expand its range of practical applications, new approaches are sought like blending with other polymers [5,6] or filling with nanofillers such as organically modified montmorillonite (OMMT) [7], multi-walled carbon nanotubes (MWCNTs) [8], nano-hydroxyapatite (HA) [9] or layered silicates [10]. Improved mechanical performance and higher thermal and barrier properties have been obtained upon addition of small amounts of nano-sized fillers into this biopolymer matrix, albeit the enhancements are still not sufficient for industrial applications.

Over recent years zinc oxide (ZnO) nanostructures have become the focus of considerable research due to their low cost, easy availability, biocompatibility and possibility of performing surface modifications with different functional groups. They possess unique chemical and physical properties like intensive ultraviolet absorption or antimicrobial activity in the pH range of 7–8 even in the absence of light, therefore are widely used for applications such as optical devices [11,12] and antimicrobials [13]. ZnO shows considerably higher antibacterial effect on *Staphylococcus aureus* than other metal oxides like MgO, TiO_2_, Al_2_O_3_, CuO or CeO_2_ [14]. In addition, these nanostructures are considered to be non-toxic and generally recognized as safe “GRAS” substances, and recent studies have reported that they do not cause any damage to the DNA of human cells [15]. They can be synthesized through various methods including hydrothermal synthesis, thermal evaporation, electrochemical decomposition, sonochemical method, sol-gel, and so forth [16]. These environmentally friendly nanomaterials possess exceptional mechanical properties, low coefficient of thermal expansion and high thermal conductivity, thus are ideal to be used as reinforcing fillers in polymer composites. In particular, ZnO nanoparticles have been shown to be very effective for enhancing the mechanical performance, barrier and/or antibacterial properties of polymers such as poly(ether ether ketone) (PEEK) [17,18], poly(lactic acid) (PLA) [19,20] and polycaprolactone (PCL) [21].

Yu *et al.* [22] reported the preparation of poly(3-hydroxybutyrate-*co*-3-hydroxyvalerate) (PHBV)/ZnO nanofibers by an electrospinning method. However, to the best of our knowledge, there is no previous work dealing with the fabrication and characterization of ZnO-reinforced PHB homopolymer, which is the main goal of the current study. For such purposes, raw ZnO nanoparticles at various loadings have been incorporated in the biopolymer matrix via a solution casting technique without the aid of surfactants or coupling agents. An extensive characterization has been carried out to analyze in detail the influence of the inorganic nanoparticles on the morphology, crystallization behaviour, thermal stability, mechanical performance, barrier, migration and antibacterial properties of these novel bionanocomposites that show great potential for food packaging applications.

## 2. Results and Discussion

### 2.1. Morphological Observations

**Figure 1:**
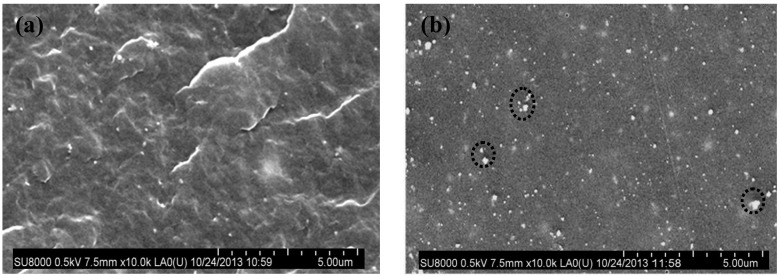
Typical SEM micrographs of PHB/ZnO nanocomposites with (**a**) 1.0 wt % and (**b**) 10.0 wt % nanoparticle content. The dashed circles on Figure 1b show small clusters of 2–4 particles.

The surface morphology of the nanocomposites was examined by SEM, and representative images of samples with 1.0 and 10.0 wt % ZnO are shown in Figure 1a,b, respectively. The white dots in the micrographs correspond to the nanoparticles that exhibit quasi-spherical shape. Random and homogeneously dispersed nanofillers can be observed in the nanocomposite with the lowest ZnO content, without any agglomerates (Figure 1a).

An even and uniform filler distribution was also found for the nanocomposites incorporating 2.0 or 5.0 wt % ZnO. The interactions between the –OH groups of the ZnO surface and the polar moieties of the biopolymer should prevent nanoparticle aggregation and improve the compatibility between the filler and matrix phases. Nevertheless, the nanocomposite with the highest loading displays not only well-dispersed nanoparticles but also a few small clusters composed of several particles (see dashed circles on Figure 1b). On the other hand, PHB/ZnO (1.0 wt %) shows a rough morphology, whilst that of the nanocomposite with 10.0 wt % ZnO is considerably smoother, a fact that is indicative of a change in the degree of crystallinity of the matrix, as will be shown from DSC analysis.

**Figure 2:**
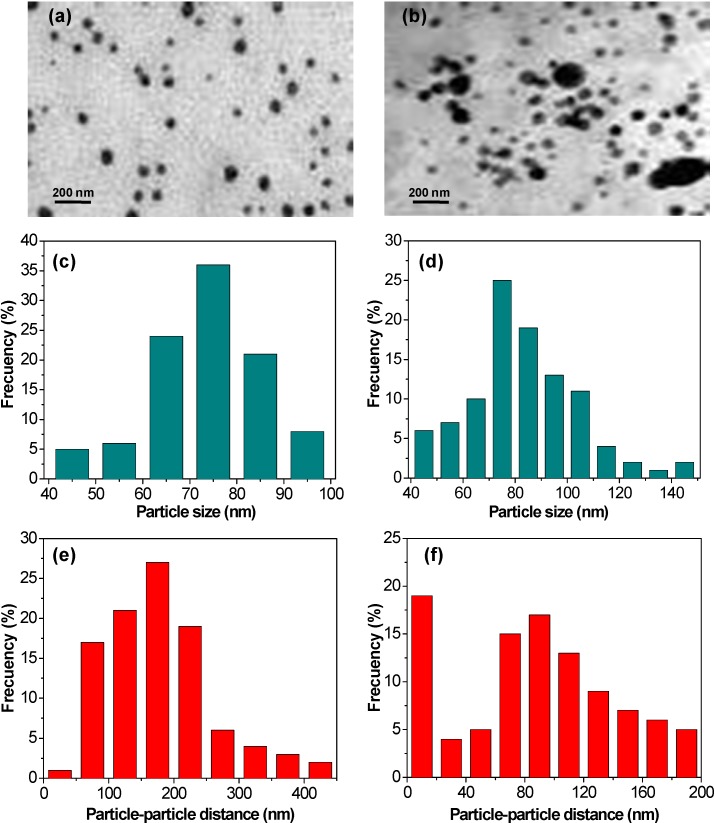
**Top**: TEM micrographs of PHB/ZnO nanocomposites with (**a**) 1.0 wt % and (**b**) 10.0 wt % nanoparticle content; **Middle** and **bottom**: Particle size and particle-particle distance distribution of ZnO nanoparticles in the nanocomposites with 1.0 wt % (**c**,**e**) and 10.0 wt % (**d**,**f**) nanofiller loading.

TEM images were recorded to further assess the state of ZnO dispersion within the matrix, and representative micrographs of the nanocomposites with the highest and the lowest loading are compared in Figure 2. Statistical analysis was performed from the TEM images to obtain information about the distribution of particle size and particle-particle distance in the nanocomposites, and the results are also shown in Figure 2.

In the sample with 1.0 wt % ZnO (Figure 2a), the nanoparticles are evenly and well distributed, without forming agglomerates. They display a narrow size distribution (Figure 2c), with diameters in the range of 40–100 nm and a mean value of 80 nm. Further, the nanofillers are quite regularly spaced, showing an average particle-particle distance of 180 nm (Figure 2e). However, at 10.0 wt % loading (Figure 2b), the nanoparticle dispersion is less uniform compared to that of the sample with lower nanofiller content. Thus, the size distribution is broader (between 40 and 150 nm) and the nanoparticle dimensions are larger, the mean diameter being 98 nm. Moreover, the average interparticle distance (75 nm) significantly decreases, resulting in increased propensity toward aggregation, since the surface hydroxyl groups of ZnO have a strong tendency to create hydrogen bonds among nanoparticles, hence flocculation of the nanofillers can occur, causing the formation of small clusters, in agreement with SEM observations. Overall, statistical analysis confirms that the dispersion of ZnO in PHB takes place as single particles at low concentrations, while both as individual particles and as small clusters at the highest loading studied. More importantly, ZnO dispersion within the biopolymer was achieved without the need for surfactants or coupling agents, making the fabrication process of these nanocomposites easier, shorter and cheaper.

### 2.2. FT-IR Study

**Figure 3:**
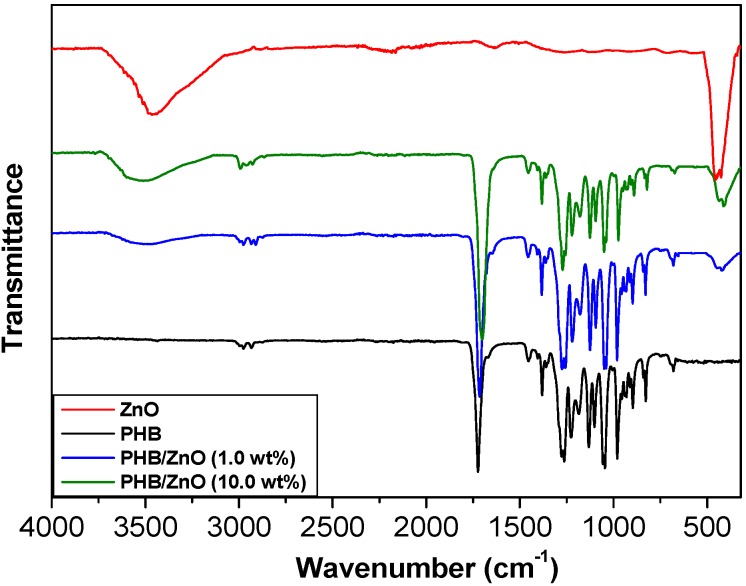
FT-IR spectra of ZnO, neat PHB and the nanocomposites with 1.0 and 10.0 wt % nanoparticle loading.

The ATR-FTIR spectra of ZnO, neat PHB and the nanocomposites with 1.0 and 10.0 wt % loading were recorded to obtain information about the nanoparticle-polymer interactions (Figure 3). The spectrum of ZnO shows a broad peak centered at 3450 cm^−1^ assigned to the stretching of hydrogen-bonded –OH groups on the nanoparticle surface. Further, an intense peak is found at 440 cm^−1^ that corresponds to the stretching of Zn–O bonds [23]. On the other hand, neat PHB displays a very strong band at 1723 cm^−1^ arising from the C=O stretching of the ester group [24]. The bands in the range of 1280–1097 cm^−1^ correspond to C–O–C stretching vibrations, and the peaks at about 2900 cm^−1^ are related to C–H stretching bands. Further, the CH_3_ asymmetric bending appears at 1457 cm^−1^, and the H–C–O in-plane bending is found at 1380 cm^−1^. The spectra of the nanocomposites show the characteristic peaks of both PHB and ZnO. The sample with 1.0 wt % loading exhibits a broadening and upshift of the peak assigned to the hydroxyl stretching, which appears centred at ~3500 cm^−1^. Such behaviour has been attributed to the change from intramolecular to intermolecular hydroxyl-hydroxyl interactions [25], and suggests hydrogen-bond formation with the carbonyl of the ester group of PHB at the expense of breaking the H-bonding among hydroxyl groups of the nanoparticles. More importantly, the carbonyl band is wider, more intense and shifts to lower wavenumber, by about 12 cm^−1^ in comparison to that of the neat polymer, indicative of the formation of hydrogen bonds with the hydroxyl moieties of the nanoparticles. An analogous behaviour has been reported for PHB/poly(styrene-*co*-hydroxystyrene) (PSHS) blends, attributed to specific H-bonding interactions between the hydroxyls of PSHS and the carbonyls of PHB [26]. Further, the peak assigned to the stretching of Zn–O bonds appears at ~430 cm^−1^, around 10 cm^−^^1^ lower than that of the bare nanoparticles, another indication of the strong ZnO-matrix interactions. Regarding the nanocomposite with 10.0 wt % loading, the shift and broadening of the bands related to the O–H and C=O stretching are considerably more pronounced. Thus, the C=O stretching appears at around 25 and 13 cm^−1^ lower than that of neat PHB and the nanocomposite with 1.0 wt %, respectively, thus corroborating the increased nanofiller-matrix interactions upon rising ZnO concentration. Besides, the peak related to Zn–O stretching shifts down to 421 cm^−1^ and is more intense than that of PHB/ZnO (1.0 wt %), in agreement with its higher nanoparticle content.

### 2.3. Thermal Stability

**Figure 4:**
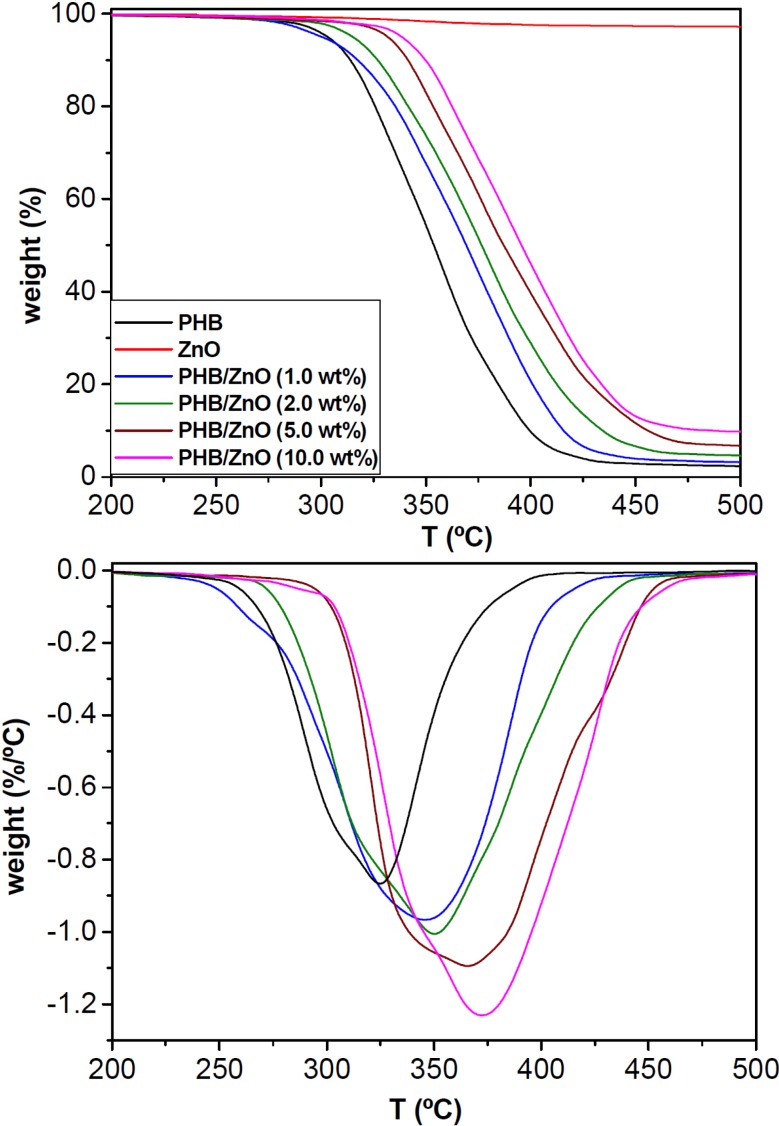
TGA (**top**) and DTG (**bottom**) curves for neat PHB, ZnO and the nanocomposites with different nanoparticle loadings.

One of the main drawbacks of PHB is its low thermal stability. Therefore, it is important to analyze the influence of ZnO nanoparticles on the thermal degradation of this biopolymer. Figure 4 shows representative TGA and DTG curves under a nitrogen atmosphere of ZnO, neat PHB and the nanocomposites with different nanoparticle loadings. The characteristic degradation temperatures, namely the initial degradation temperature at 2% weight loss (*T*_i_), the temperature of 10% weight loss (*T*_10_) and the temperature of maximum rate of degradation (*T*_max_) of all the nanocomposites are collected in Table 1. The bare nanoparticles exhibit a very small weight loss (~0.8 wt %) below 300 °C, probably due to the removal of physically and chemically adsorbed water on their surface. Regarding the polymeric samples, neat PHB displays a single degradation stage that starts at ~290 °C and shows the maximum rate at around 330 °C (Table 1). The degradation occurs via random chain scission mechanism, producing shorter chain fragments with carboxylic terminal groups and with crotonic acid as one of the characteristic by-products [27]. Analogously, a one step degradation process is observed for the nanocomposites, albeit shifted to higher temperatures, pointing out the thermal stabilization effect caused by the nanoparticles. Thus, *T*_i_ increases by 8, 21 and 24 °C upon addition of 2.0, 5.0, and 10.0 wt % ZnO, respectively. A similar trend is found for *T*_10_ and *T*_max_, with increments by up to 34 and 42 °C, respectively, at the highest loading tested (Table 1). These results demonstrate that the incorporation of ZnO improves the heat resistance of PHB matrix, ascribed to the barrier effect of the nanoparticles that effectively hinder the transport of decomposition products from the bulk of the matrix to the gas phase. Moreover, the high thermal conductivity of ZnO [28] should facilitate heat dissipation within the composite, thus resulting in enhanced thermal stability.

**Table 1:** Thermal parameters obtained from DSC and TGA analysis of PHB/ZnO nanocomposites.

ZnO (wt %)	*T*_c_ (°C)	*T*_m_ (°C)	*X*_c_ (%)	*T*_i_ (°C)	*T*_10_ (°C)	*T*_max_ (°C)	CR (%)
0	76.3	163/174	52.5	291.2	314.7	329.6	1.7
1.0	93.4	165/173s^a^	56.9	288.5	317.2	345.5	2.6
2.0	101.2	166/171s^a^	59.4	299.0	324.9	348.3	3.8
5.0	106.0	167/171s^a^	63.2	312.2	341.6	365.7	5.9
10.0	105.6	169	61.8	314.8	347.9	372.3	10.2

*T*_c_ and *T*_m_: crystallization and melting temperature, respectively; s^a^: shoulder; *X*_c_: degree of crystallinity; *T*_i_: initial degradation temperature at 2% weight loss; *T*_10_: temperature for 10% weight loss; *T*_max_: temperature of maximum rate of weight loss; CR: Char Residue.

Further, a gradual rise in the char residue is observed with increasing ZnO content, indicating that the thermal decomposition of the matrix is retarded in the nanocomposites. The nanoparticles act as insulator and mass transport barriers that obstruct the escape of volatile products generated during the degradation process. It is important to note that the increments in *T*_i_ and *T*_max_ obtained upon addition of ZnO are comparable to those reported for MWCNT-reinforced PHB nanocomposites [8] and higher than those found with the incorporation of similar amounts of OMMT [7], cellulose nanowhiskers (CNWs) [29] or Ag_2_S nanoparticles [30].

### 2.4. Crystallization and Melting Behaviour

**Figure 5:**
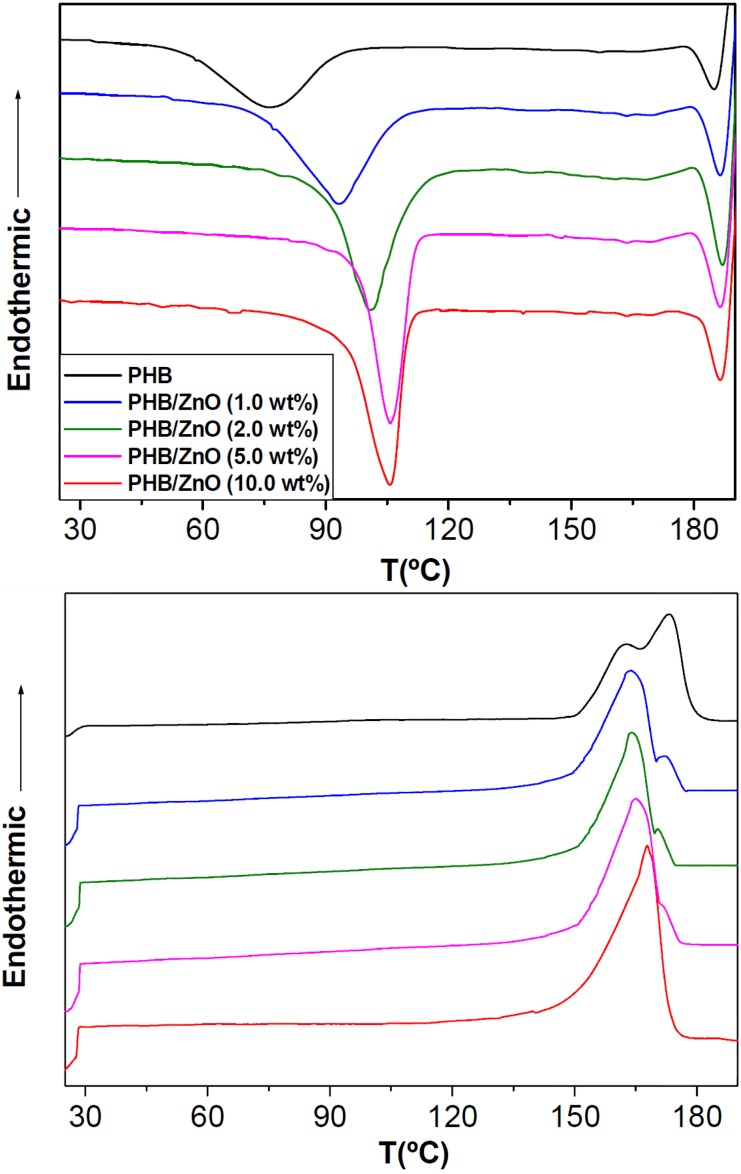
Non-isothermal DSC cooling (**top**) and second heating (**bottom**) scans of PHB and the nanocomposites at a rate of 10 °C/min.

The study of the crystallization and melting behaviour of polymer composites is of great interest since it influences not only the crystalline structure and morphology but also the macroscopic properties of the materials. To evaluate these effects, neat PHB and its nanocomposites were subjected to DSC analysis under non-isothermal conditions, and their thermograms are shown in Figure 5. The calorimetric parameters obtained from DSC curves are summarized in Table 1. Neat PHB has a crystallization peak temperature (*T*_c_) of ~76 °C, which shifts to higher temperature in the presence of the nanoparticles. This increase is more pronounced at low ZnO loadings (about 17 °C rise at only 1.0 wt %), and becomes almost constant at higher concentrations (≥5.0 wt %), the maximum *T*_c_ increment being around 30 °C. These results indicate that ZnO nanoparticles accelerate the crystallization of PHB due to heterogeneous nucleation effect. Further, the crystallization peak sharpens in the nanocomposites, demonstrating that these nanofillers effectively act as nucleating agents and increase the overall crystallization rate of PHB.

A similar trend is found within the level of crystallinity (*X*_c_), which increases from ~53% for the neat matrix to about 63% for the nanocomposite with 5.0 wt % ZnO, while remains merely unchanged at higher loading. At 10.0 wt %, albeit the higher amount of nanoparticles should supply a larger nucleation surface, the strong interactions between the –OH groups of the ZnO and the C=O of the ester groups of PHB might impose significant restrictions on polymer chain diffusion and crystal growth, and the overall result is a negligible change in *T*_c_ and *X*_c_. It is worthy to note that analogous nucleating roles of PHB crystallization have been previously reported for other nanofillers like MWCNTs [8] or HA [9], albeit the nucleating effect of ZnO nanoparticles is stronger, probably due to their more homogenous dispersion, hence a larger number of nucleating centers and consequently, higher crystallization temperature. In contrast, a decrease in T_c_ has been reported for PHB nanocomposites reinforced with OMMT [7] or Ag_2_S [30], attributed to chain scission degradation that causes a decrease in the molecular weight and chain length of the matrix. The higher crystallinity attained in ZnO-reinforced PHB materials is a key factor to enhance their mechanical performance. On the other hand, in PHBV/ZnO nanofibers fabricated by an electrospinning method [22], the nanoparticles were found to slow down the crystallization process of the copolymer matrix, probably related to the preferential location of ZnO in certain regions of the fibers; this demonstrates the strong influence of the processing method on the state of nanofiller dispersion and location within the matrix, hence on the final properties of the nanocomposites.

Regarding the heating scans obtained after dynamic crystallization (lower part of Figure 5), it is found that virgin PHB exhibits double melting behaviour that can be explained by the melting, recrystallization, and remelting model [31]. The first endothermic peak at 163 °C (*T*_m1_) corresponds to the melting of the crystals formed during the non-isothermal crystallization from the melt at a constant cooling rate, whereas the second at 174 °C (*T*_m2_) arises from the melting of crystals formed through recrystallization and reorganization during the DSC heating process. The nanocomposites with lower ZnO content also show double melting behaviour, with a main peak at lower temperature (*T*_m1_ = 165 or 167 °C at 1.0 or 5.0 wt %, respectively), and a small shoulder at higher temperature (*T*_m2_ = 173 or 171 °C, respectively). A slight increase in *T*_m1_ is found with increasing nanofiller loading (Table 1), together with a narrowing of the peak, in agreement with the increase in crystallinity found from the cooling thermograms. The reduced area of the second endothermic peak in these nanocomposites in comparison to that of PHB indicates that ZnO nanoparticles restrict the recrystallization of the matrix crystals. An analogous behaviour of recrystallization constraint has been previously reported for PHB nanocomposites reinforced with MWCNTs [32]. On the other hand, the composite with the highest loading displays a single melting peak at about 169 °C. The disappearance of double endothermic peaks provides further evidence for the highly efficient nucleation activity of ZnO that improves the crystallization of this biopolymer. Therefore, the crystals in the nanocomposites are more perfect and stable than those of neat PHB. Further, the mobility restriction arising from the strong nanoparticle-matrix interactions is another important factor that probably contributes to hamper the recrystallization of PHB in the nanocomposites.

### 2.5. Dynamic Mechanical Study

**Figure 6:**
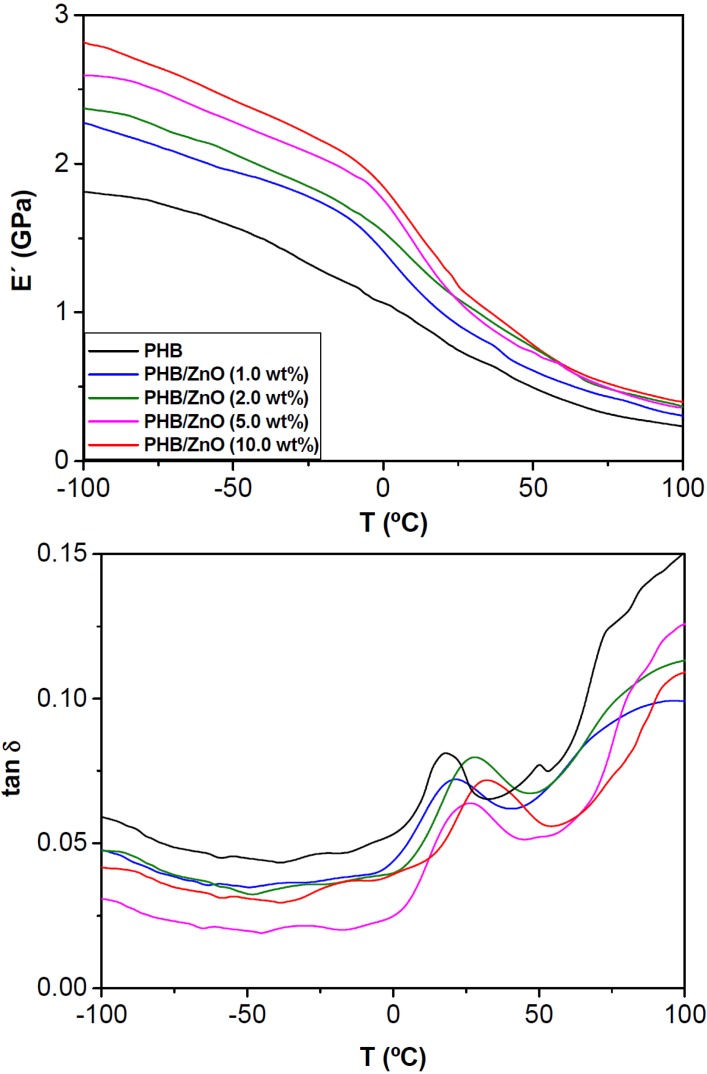
Storage modulus *E*' (**top**) and tan δ (**bottom**) as a function of temperature for neat PHB and the nanocomposites with different content of ZnO nanoparticles.

A common reason for adding inorganic nanofillers to biopolymers is to increase the mechanical properties of the resultant composites. In particular, food packaging materials should have enough stiffness and strength to resist handling damage. Moreover, containers are commercially used below room temperature (*i.e.*, at frozen food storage temperatures from −30 to −18 °C or refrigeration conditions between 3–6 °C), hence it is important to assess their mechanical performance under such conditions. The temperature dependence of the storage modulus (*E*') and loss factor (tan δ) for neat PHB and the composites with different ZnO contents was evaluated by DMA, and the results are shown in Figure 6. *E*' values at different temperatures are listed in Table 2. At temperatures below the glass transition (*T*_g_), *E*' rises progressively upon increasing ZnO concentration; thus, at 25 °C, a maximum value of ~1.2 GPa (about 51% increase compared to that of neat PHB) is attained, demonstrating the strong reinforcing effect of these nanoparticles. Indeed, the storage modulus reveals the capability of a material to store mechanical energy without dissipation; the higher the storage modulus, the stiffer the material is. This improvement should also be related to the increase in the crystallinity of the matrix, since the crystalline regions are known to enhance the modulus of semicrystalline polymers, combined with a strong filler-matrix interfacial adhesion due to H-bonding interactions between the –OH groups of ZnO and the carbonyl of the ester groups of PHB, as mentioned previously. At temperatures above *T*_g_ (Table 2), the differences between *E*' of each composite and the matrix are in general less significant, indicating that the stiffening effect is more pronounced below the matrix softening point.

**Table 2:** DMA data for neat PHB and the nanocomposites with different ZnO loadings.

ZnO (wt %)	*T*_g_ (°C)	*E*'_−100__°C_ (GPa)	*E*'_25__°C_ (GPa)	*E*'_100__°C_ (GPa)	10^2^ tan δ_max_ (a.u.)	FWHM (°C)	tan δ_area_ (a.u.)
0	17.9	1.81	0.78	0.24	8.11	13.9	0.79
1.0	22.1	2.27	0.93	0.30	7.23	19.2	1.06
2.0	28.4	2.39	1.11	0.37	7.98	22.9	1.13
5.0	27.7	2.59	1.11	0.36	6.35	26.8	1.18
10.0	32.6	2.78	1.18	0.39	7.19	24.5	1.11

*E*': storage modulus; *T*_g_: glass transition temperature; tan δ_max_: tan δ maximum value; FWHM: full-width at half maximum of tan δ peak; tan δ_area_: area under tan δ peak.

The damping factor (tan δ, ratio of the loss to storage modulus) gives information about the energy lost in a system due to deformation. As also depicted in Figure 6, the plot of tan δ as a function of temperature for PHB shows an intense maximum named α relaxation that corresponds to the *T*_g_ at about 18 °C. The low temperature relaxation (ã) at around −100 °C, related to the presence of absorbed water in the amorphous domains, as well as the α_c_ relaxation in the range 80–130 °C attributed to motions of the crystalline phase can only be inferred in the DMA spectrum of this biopolymer [33]. With regard to tan δ data for the different compositions (see Table 2), it is evident that the introduction of ZnO results in a shift of *T*_g_ toward higher temperature; this suggests restrained mobility of the polymer chains in the presence of the quasi-spherical nanoparticles. In particular, *T*_g_ increases by nearly 15 °C for the nanocomposite with the highest ZnO content as compared to that of pure PHB. Since the glass transition is associated with the mobility of chain segments in the amorphous regions, a plausible explanation is that those segments in the vicinity of the nanoparticles are less mobile, because these restrict rotational motion within the chains, and therefore lead to a raise in *T*_g_. Such behaviour has been reported for PHB composites reinforced with HA nanoparticles [9].

It is also noteworthy from Figure 6 that the maximum value of tan δ is lower for the nanocomposites compared to that of the neat matrix. A high value of tan δ typically implies imperfections in the elasticity of a system. Therefore, the lower tan δ in the composites suggests that when the stress is removed, the energy stored in deforming the material is recovered more quickly compared to the unfilled polymer. In addition, a certain broadening of tan δ is observed in the presence of the nanoparticles (Table 2). This effect could arise from a more heterogeneous amorphous phase in the nanocomposites compared to that of the neat matrix, and has also been interpreted as enhanced nanoparticle-matrix interactions [34]. Another interesting parameter is the area under tan δ peak (Table 2), since it is representative of the energy dissipated in the viscoelastic relaxations, hence it is indicative of the impact toughness of the material. All the nanocomposites exhibit larger area than the neat polyester, indicating that these nanoparticles improve the matrix stiffness without a detriment in its ability to dissipate energy. Overall, experimental results demonstrate that PHB nanocomposites display good rigidity under normal freezing and refrigeration conditions, showing considerably improved performance than that of the neat biopolymer.

### 2.6. Tensile Properties

**Figure 7:**
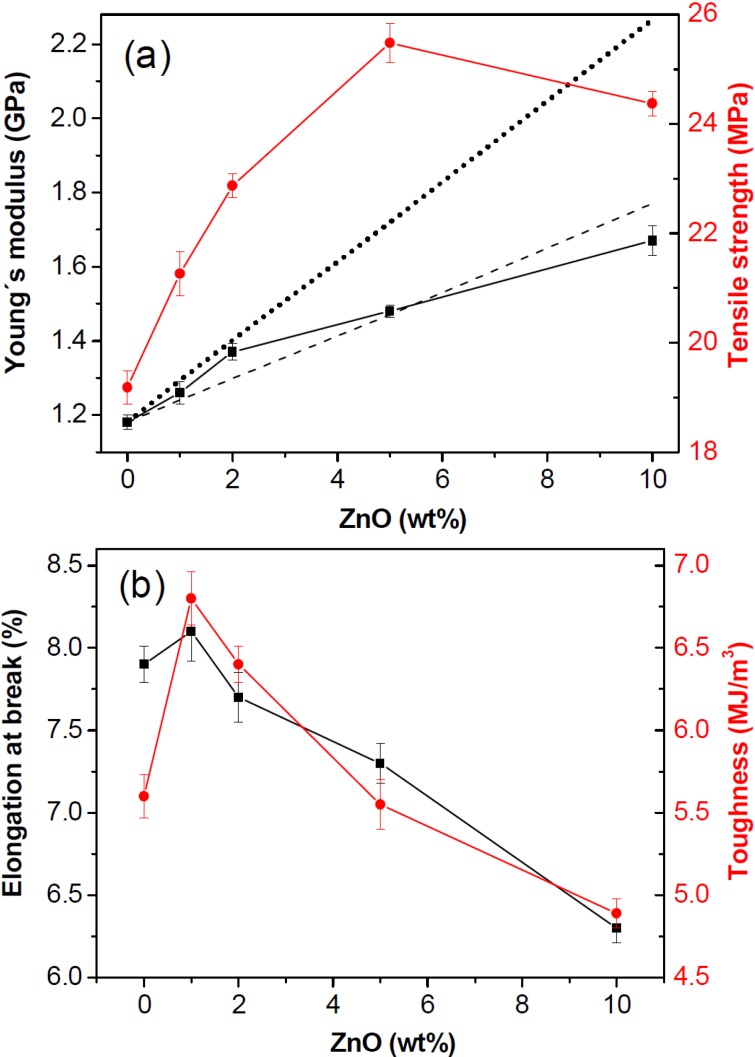
Tensile properties of PHB and PHB/ZnO nanocomposites with different nanoparticle loadings: (**a**) Young’s modulus and tensile strength; (**b**) strain at break and toughness. The dotted and dashed lines in Figure 7a correspond to the predictions according to the Krenchel’s rule of mixtures and Einstein’s equation, respectively (see explanation in the text).

The room temperature static mechanical properties were evaluated by tensile tests, and the values of Young’s modulus (*E*), tensile strength (σ_y_), elongation at break (ε_b_) and toughness (*T*) derived from the stress-strain curves of the different composites are displayed in Figure 7. Neat PHB shows a Young’s modulus of ~1.2 GPa. The addition of ZnO nanoparticles leads to a gradual rise in E (Figure 7a), showing a maximum augment of 43% at 10.0 wt %. The mechanical properties of polymer composites are affected by many factors, the most important being the state of dispersion of the filler, the degree of crystallinity of the matrix and the filler-matrix interactions. Thus, the strong *E* enhancement attained in these nanocomposites is attributed to the combination of fairly good nanoparticle dispersion, a strong interfacial adhesion between the phases through interactions via H-bonding and the increase in the crystallinity of PHB. A very similar trend is found for the tensile strength, with a maximum increase of 32% at 5.0 wt %, albeit it decreases slightly at higher loadings. It is worthy to note that the reinforcement effect found for these inorganic nanoparticles is more effective than that attained with similar amounts of other nanofillers such as OMMT [7] or MWCNTs [35], demonstrating the suitability of the approach used in this work to improve the mechanical performance of biodegradable polyesters.

Taking into account the reported Young’s modulus for ZnO [36], the theoretical E values for the nanocomposites were estimated by the Krenchel’s rule of mixtures for discontinuous reinforcement [37], which can be written as:

*E_c_* = (η*E_f_* − *E_m_*)*V_f_* + *E_m_* (1)

where *E_c_*, *E_f_* and *E_m_* are the tensile modulus of the composite, filler and matrix, respectively, *V_f_* the filler volume fraction and η the strengthening coefficient that is assumed to be 1/5 for randomly oriented fillers. The results from Equation (1) are plotted in Figure 7a as a dotted line. Experimental Young’s moduli of PHB/ZnO nanocomposites deviate strongly from the theoretical calculations and are systematically below the predicted values, which suggests that this generic model is not suitable for the current system. A more accurate estimation can be obtained based on Einstein’s equation for prediction of *E* of rigid particulate composites, which can be expressed as [38]:

*E_c_* = *E_m_* (1 + 2.5*V_f_*) (2)

This simple approach, originally derived for the effective shear viscosity of dilute suspensions of rigid spheres, is valid only at low filler concentrations and assumes perfect filler-matrix adhesion and optimal dispersion of individual particles. Theoretical data derived from Equation (2) (see dashed line in Figure 7a) are in good agreement with the experimental measurements (differences < 7%). The experimental value is lower than the prediction for the nanocomposite with 10.0 wt % ZnO, likely related to the presence of small nanoparticle clusters.

On the other hand, the strain at break (Figure 7b) remains merely unchanged up to 2.0 wt % ZnO and then drops moderately, by about 20% at the highest concentration tested. In the composites with higher loadings the nanoparticles restrict the ductile flow of the polymer chains, which is reflected in lower ε*_b_* values. Nevertheless, for similar nanofiller contents, the fall in tensile strain found for OMMT [7] or MWCNT-reinforced PHB [35] nanocomposites is considerably stronger (*i.e.*, about 50% at 3.0 wt % MWCNT), related to severe nanofiller aggregation. Figure 7b also depicts the toughness of the nanocomposites measured as the area under the tensile curve. The toughness of PHB increases with the addition of nanoparticle contents ≤2.0 wt %, showing a maximum improvement of ~21% at the lowest concentration tested. The nanocomposite with 5.0 wt % displays similar toughness to that of the neat biopolymer, while that with the highest ZnO loading shows a small decrement of around 12%. These results indicate that the incorporation of small amounts of these nanoparticles improves the ability of PHB to absorb energy during the deformation process.

### 2.7. Impact Strength

**Figure 8:**
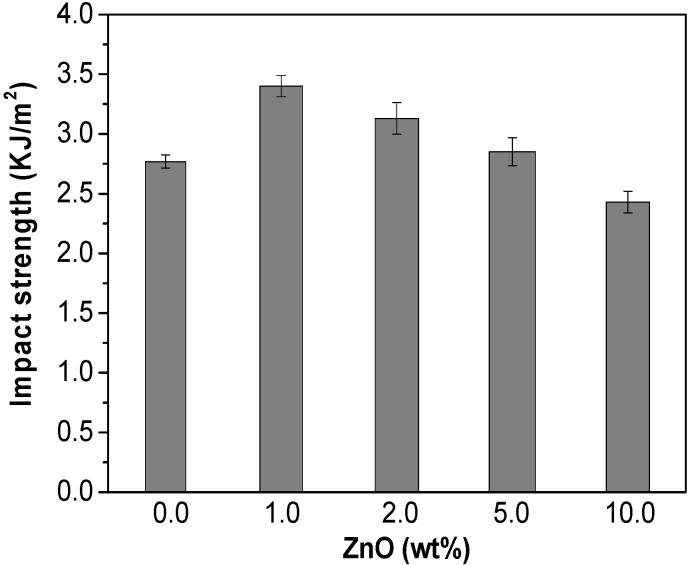
Charpy notched impact strength of PHB and its nanocomposites.

Neat PHB has a high crystallinity (about 52%), which leads to an inherent brittleness and poor impact resistance, drawbacks that limit its application as food packaging material. Therefore, it is of great interest to improve the impact strength of this thermoplastic by the addition of nanofillers. The results from Charpy notched impact strength measurements of PHB and the ZnO-reinforced nanocomposites are plotted in Figure 8. The incorporation of 1.0 wt % ZnO leads to a noticeable improvement in the impact resistance of the polymer, by around 26%, consistent with the larger extent of plastic deformation of this nanocomposite as revealed by tensile test. An enhancement is also observed for the composites with 2.0 and 5.0 wt % ZnO, whereas that with the highest nanoparticle concentration shows a slightly lower value (~10% reduction). This dependence of the impact strength on the ZnO content was already anticipated from the analysis of the areas under the tensile curves, and the results obtained by the two techniques display very similar trend.

It has been widely reported [39] that the shape, size, state of dispersion of the filler and its interfacial adhesion with the matrix have strong influence on the rate of energy absorption, hence on the impact properties of polymer composites. The toughness of PHB improves considerably by the addition of low ZnO contents, ascribed to a very homogeneous nanofiller dispersion that minimizes the stress concentration nuclei, as well as an enhanced interfacial adhesion between the composite phases via H-bonding that should provide an effective barrier for pinning and bifurcation of the advancing cracks. Nonetheless, in the composite with the highest ZnO loading, the presence of small nanoparticle clusters might nucleate a few cracks and/or promote the formation of little dimples, leading to a slight reduction in ductility. Therefore, ZnO loadings in the range of 1–5 wt % are found to be effective for enhancing the impact resistance of PHB.

### 2.8. Barrier and Migration Properties

A critical issue in food packaging is that of migration and permeability. Thus, one of the main goals when adding nanofillers to biopolymer matrices is to improve their barrier properties to gases, vapours and organic compounds. Water vapour and oxygen are the two main permeants studied in packaging applications, because they may transfer from the internal or external environment through the composite package wall, resulting in a continuous change in product quality and shelf-life [2]. The specific barrier requirements depend on the product characteristics and the intended end-use application.

**Figure 9:**
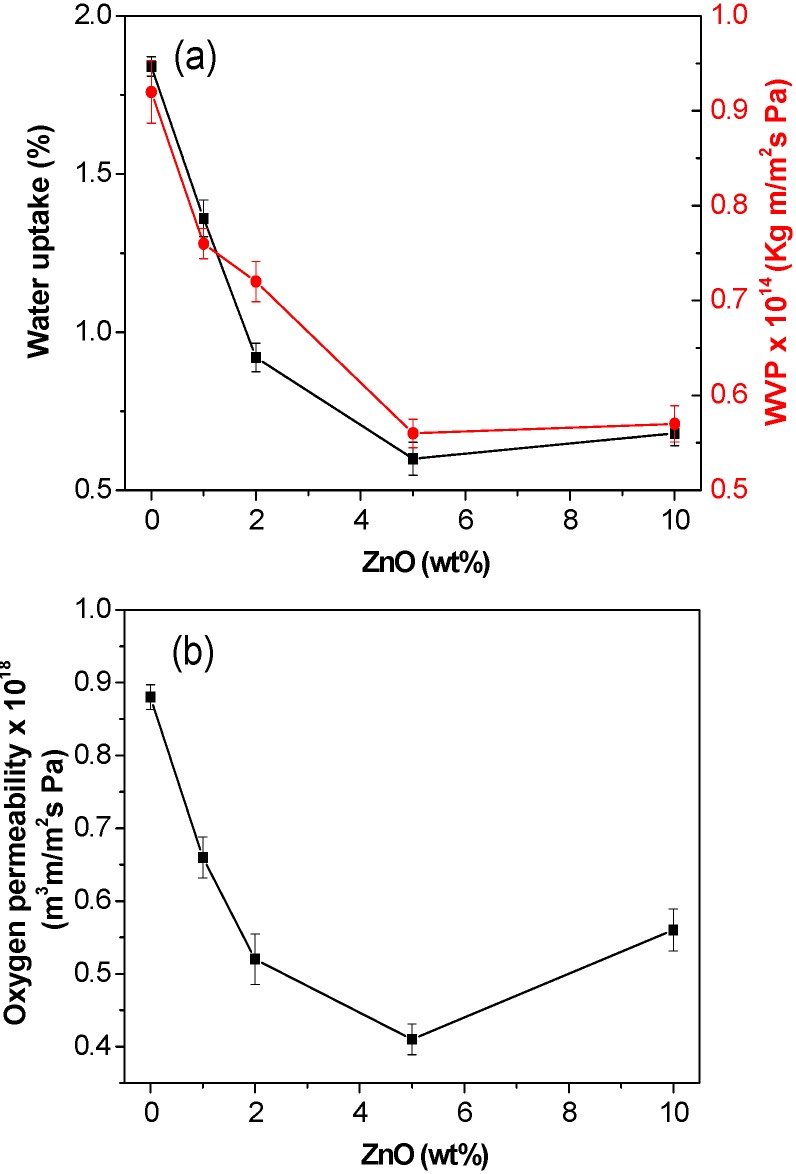
Barrier properties of PHB/ZnO nanocomposites as a function of ZnO content: (**a**) Water uptake and water vapour permeability (WVP); (**b**) Oxygen permeability.

In this study, the water uptake and water vapour permeability (WVP, amount of water vapour that permeates per unit of area and time in a packaging material) of the nanocomposites were tested, and the results are shown in Figure 9a. Clearly, both parameters decrease with increasing ZnO content, by up to 66% and 38% at 5.0 wt % nanoparticle content, respectively, which demonstrates improved barrier properties against water and water vapour for the nanocomposites in comparison to the neat biopolymer. This enhanced behaviour could be related to the more perfect crystalline structure of the matrix, as revealed by DSC analysis. Thus, PHB/ZnO samples have reduced fraction of amorphous phase through which water molecules can permeate. Moreover, the higher degree of crystallinity increases the tortuosity of the transport path [40]. However, the water uptake and WVP slightly increase upon addition of 10.0 wt % ZnO, probably due to the formation of a few nanoparticle clusters.

Regarding the oxygen permeability of the nanocomposites (Figure 9b), a noticeable reduction is also observed as the ZnO content rises, showing a minimum (about 53% decrement as compared to that of neat PHB) at a critical nanofiller concentration of 5.0 wt %. This improved barrier performance should be related to the homogeneous ZnO dispersion, its strong interfacial adhesion with the polyester matrix that causes chain immobilization combined with the increase in the degree of crystallinity of the matrix. Further, it has been demonstrated that ZnO nanoparticles reduce the oxygen permeability of polymer nanocomposites not only by creating a tortuous path but also via gas adsorption onto their surface [21]. However, the oxygen permeability of the composite with the highest loading is higher than that with 5.0 wt % ZnO, probably because nanofiller aggregation starts to take place, resulting in the formation of preferential paths for the permeants to diffuse faster, hence reduced barrier performance. A qualitatively similar trend has been reported for PHB nanocomposites filled with nanoclay [41], where composites containing 5.0 wt % nanofiller content exhibited the lowest oxygen permeability value. Results demonstrate that the incorporation of ZnO to PHB matrix has a positive effect on the gas barrier properties, rendering improved materials for packaging oxygen- and/or moisture-sensitive products.

**Figure 10:**
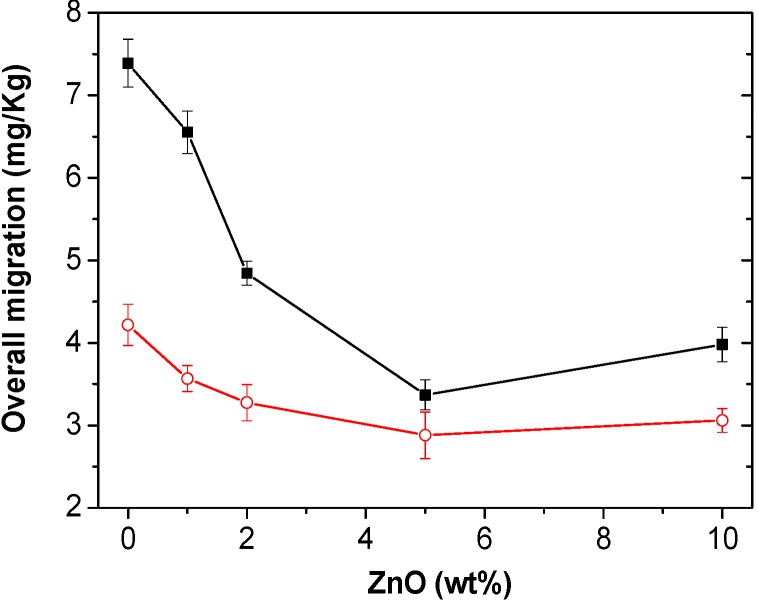
Overall migration data in ethanol 10% (*v*/*v*) (solid squares) and isooctane (open circles) for PHB and its bionanocomposites.

Migration is the quantity of packaging material, principally additives, which can be transferred to foodstuff. Generally, migration has been regarded as a negative issue since many substances represent a danger to human health and/or modify the food composition. Overall migration tests with non-polar and polar simulants were carried out to investigate the total amount of non-volatile substances that might migrate into foodstuff from PHB and its nanocomposites. The results from experiments performed in isooctane and 10% (*v*/*v*) ethanol are shown in Figure 10. It is worthy to note that, for both simulants, the maximum migration levels are well below the migration limits for food contact materials (60 mg/kg or 10 mg/dm^2^ of simulant) established by current legislation (Commission Directive 2002/72/EC). The residues found may be monomers released in the PHB synthesis or additives used during the polymer manufacturing process [42]. More importantly, increasing the ZnO content remarkably reduces the migration in ethanol 10% (*v*/*v*), by up 54% at 5.0 wt % loading, which should be related to the enhanced nanofiller-matrix adhesion, as discussed previously.

A decreasing trend is also found for migration in isooctane, the maximum reduction being ~29% at the same critical ZnO concentration. In this case, the migration levels are smaller compared to those in ethanol, in agreement with the results reported for other composites based on polyester matrices such as PLA [43]. The different migration values found for both simulants most likely arise from polarity and solubility differences between the polymer, nanofiller, migrants and the food simulant, since such thermodynamic properties play a key role on the overall migration. Regarding the nanocomposite with 10.0 wt % ZnO, a slight increment in both migration levels is detected, probably related to the presence of small nanoparticle clusters that provoke a reduction in matrix homogeneity and cohesion, which has a negative effect on the migration properties. Overall, the results suggest that the developed biodegradable nanocomposites could be used as containers for food and beverage products under different storage conditions.

### 2.9. Antibacterial Properties

The incorporation of antimicrobial compounds into food packaging materials has recently received considerable attention [1]. Composites with antimicrobial activity restrict the growth of pathogenic and spoilage microorganisms. Metal nanoparticles (Ag, Cu, Au), metal oxide nanomaterials (TiO_2_, ZnO, MgO), and carbon nanotubes are the most commonly employed nanofillers to develop antimicrobial action. Albeit Ag has been traditionally used as an antimicrobial agent in food and beverage storage applications, ZnO nanoparticles are expected to provide a more affordable and safe food packaging solution in the future.

**Figure 11:**
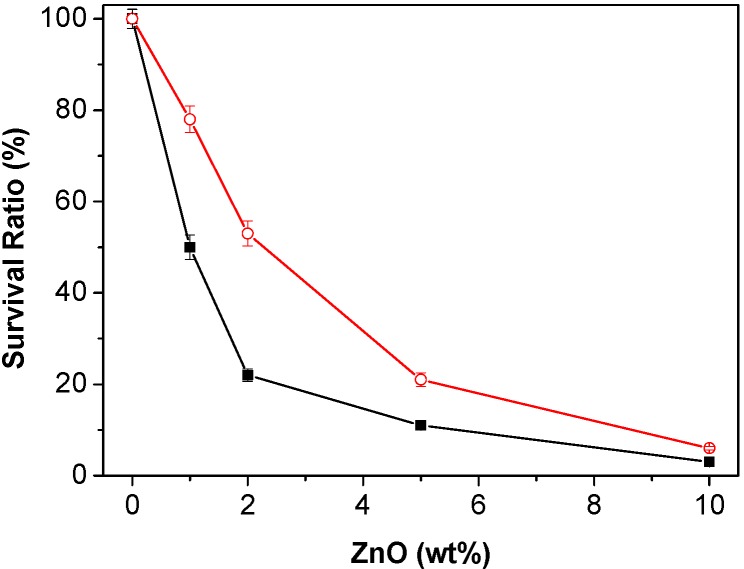
Effect of PHB/ZnO nanocomposites on the survival ratio of *E. coli* (solid squares) and *S. aureus* (open circles).

In this regard, the antibacterial action of neat PHB nanocomposites was tested against two human pathogen bacteria: *E. coli* (Gram-negative) and *S. aureus* (Gram-positive), and the results are shown in Figure 11. As can be observed, the survival ratio of both bacteria decreases exponentially with increasing ZnO content, and the best antibacterial activity (about 97% and 94% growth inhibition for *E. coli* and *S. aureus*, respectively) is attained with 10.0 wt % loading. This strong bacterial inactivation could be explained in terms of the larger nanoparticle effective surface area, hence enhanced surface reactivity. Interestingly, the antibacterial effect of PHB nanocomposites on *E. coli* is systematically stronger than on *S. aureus*, in agreement with previous studies [44] that reported more effective action of ZnO nanoparticles against Gram-negative bacteria, and that composites with high ZnO contents can drastically inhibit the growth of the microorganisms. The different behaviour against the two types of bacteria has been attributed to structural and chemical compositional differences of the cell surfaces [45]. Thus, Gram-positive bacteria usually have one cytoplasmic membrane and a thick wall composed of multilayers of peptidoglycan, while the Gram-negative have a more complex cell wall structure, with a layer of peptidoglycan between the outer membrane and the cytoplasmic membrane. Moreover, the difference could be related to the tendency of *S. aureus* to form aggregates that would protect more internal cells from small doses of antimicrobial products [19]. For both bacteria, the antimicrobial activity has been ascribed to the damage of the cell membranes, which leads to leakage of cell contents and cell death. Although the exact mechanism of action is still unknown, the production of H_2_O_2_ (a strong oxidizing agent harmful to the cells of living organisms) from the ZnO surface has been considered as the key factor of antibacterial activity of ZnO-reinforced nanocomposites [46], and the different action against *E. coli* and *S. aureus* probably arises from the different sensitivities towards the H_2_O_2_ generated. Overall, results demonstrate the great potential of the developed nanocomposites to prevent microbial contamination, which is of great interest for food packaging applications.

## 3. Experimental Section

### 3.1. Materials and Preparation of the Nanocomposites

Poly(3-hydroxybutyrate) (PHB), Biomer P226, was obtained from Biomer Ltd. (Krailling, Germany). It presents the following physical characteristics: *M*_W_ ~ 80.000 g/moL; *T*_g_ ~ 20 °C, *T*_m_ ~ 175 °C, *d*_25__°C_ = 1.25 g/cm^3^. The polymer was dried in an oven at 60 °C for 20 h before use. Zinc oxide nanopowder, <100 nm particle size and specific surface area in the range of 15–25 m^2^/g, was supplied by Sigma-Aldrich (Madrid, Spain). The surface hydroxyl concentration of ZnO was determined by titration with triethyl aluminium (TEA) [47]. To remove physisorbed water, the nanoparticles were previously dehydrated at 200 °C for 8 h under vacuum. The titration was carried out in triplicate, leading to an average hydroxyl group concentration of 0.25 mmol/g. Assuming that the hydroxyl groups are randomly distributed on the ZnO surface and taking into account a mean specific nanoparticle surface area of 20 m^2^/g, the hydroxyl surface density was estimated to be 7.5 OH/nm^2^. This low value should be related to the synthesis process of the nanoparticles, since commercially available nanopowders are frequently subjected to high temperatures during their synthesis that can remove part of the surface hydroxyl groups [48]. Chloroform (CHCl_3_) was also purchased from Sigma-Aldrich (Madrid, Spain) and used as received. The solution casting technique was used to prepare pure PHB films.

Regarding the nanocomposites, a combination of ultrasonication with the solution casting method was employed to improve the nanofiller dispersion within the matrix. Firstly, a certain amount of ZnO was dispersed in chloroform by ultrasonication at 100 W for 30 min. Subsequently, the PHB powder was dissolved at 50 °C in the nanoparticle dispersion and the mixture was sonicated again at 20 W for 20 min. The resulting blend was poured into a glass Petri dish to evaporate the chloroform at room temperature and finally dried under vacuum for 48 h to remove the residual solvent. The final ZnO concentrations in the nanocomposites were 1.0, 2.0, 5.0 and 10.0 wt %. To confirm the absence of reaction between PHB and ZnO during the preparation of the nanocomposites, the polymer molecular weight was determined by gel permeation chromatography (GPC), and the results showed no change in the *M*_W_ as compared to that of pure PHB.

### 3.2. Characterization Techniques

The morphology of the nanocomposites was examined by scanning electron microscopy (SEM) with a SU8000 Hitachi microscope (Minato-ku, Tokyo, Japan) applying an acceleration voltage of 2.0 kV. Samples were cryo-fractured in liquid nitrogen and then coated with a ~5 nm Cr overlayer to avoid charge accumulation during electron irradiation. Transmission electron microscopy (TEM) images were obtained with a Philips Tecnai 20 FEG (LaB_6_ filament) (Philips, Marconilaan, The Netherlands) electron microscope fitted with an EDAX detector operating at 200 kV. Ultra-thin sections of the composites were cut using a diamond knife and a Reichert Ultracut-S ultramicrotome (Leeds Precision Instruments, Minneapolis, MN, USA) equipped with a FCS cryo-device and placed onto copper grids.

The attenuated total reflectance FT-IR spectra were recorded at room temperature on a Perkin-Elmer Spectrum One spectrometer (PerkinElmer Inc., Massachusetts, MA, USA) equipped with a Universal ATR sampling accessory (diamond crystal) and a red laser excitation source (632.8 nm). Four scans were collected for each sample in the wavelength range between 4000 and 400 cm^−1^. To improve the signal-to-noise ratios, spectra were recorded with an incident laser power of 1 mW and a resolution of 4 cm^−1^.

The thermal stability of the composites was analyzed by thermogravimetric analysis (TGA) using a TA Instruments Q50 thermobalance (TA Instruments Ltd., Hertfordshire, UK) at a heating rate of 10 °C/min. Prior to the measurements, samples were dried overnight at 50 °C. The temperature was scanned from room temperature to 500 °C under a nitrogen atmosphere. Experiments were carried out on samples with an average mass of 20 mg, with a purge gas flow rate of 60 mL/min.

Dynamic differential scanning calorimetry (DSC) experiments were conducted on a Mettler DSC 30 (Mettler-Toledo, Lancashire, UK) with a TC15 TA controller under a nitrogen atmosphere. Samples with an average mass of 12 mg were melted at 190 °C and kept at this temperature for 5 min to erase the thermal history of the material. Subsequently, they were cooled to 25 °C and reheated to 190 °C, all the steps at a constant rate of 10 °C/min. The transition temperatures were taken as the peak maximum or minimum in the calorimetric curves. The degree of crystallinity was calculated from the normalized peak enthalpies following the equation:

*X_m_* = *∆H_m_*_,*PHB*_/(*∆H°_m_*_,*PHB*_ × *w_PHB_*) (3)

where ∆*H_m_*_,*PHB*_ is the apparent melting enthalpy of PHB, *w_PHB_* is the weight fraction of PHB in the composites and *∆H°_m_*_,*PHB*_ is the theoretical value of enthalpy for a 100% crystalline sample (146 J/g) [49].

The dynamic mechanical performance was studied using a Mettler DMA861 (Mettler-Toledo, Lancashire, UK) dynamic mechanical analyzer. Measurements were carried out in the tensile mode on rectangular shaped bars, in the temperature range between −100 and 100 °C at a heating rate of 2 °C/min and frequency of 1 Hz. A dynamic force of 6 N was used oscillating at fixed frequency and amplitude of 30 μm.

Tensile tests were carried out according to the ASTM D 638-03 standard [50] on a servo-hydraulic testing machine (type MTS 858) placed into an environmental chamber at 23 °C and 50% relative humidity (RH), with a crosshead speed of 1 mm/min and a load cell of 100 kN. All the samples were conditioned for 24 h before the measurements. Five specimens for each type of nanocomposite were tested to check for repeatability.

Charpy notched impact strength tests were carried out on a CEAST Fractovis dart impact tester (Massachusetts, MA, USA), using a hammer mass that impacted with an energy of 7.10 J on notched specimen bars, according to the ASTM D 6110-10 standard [51]. Measurements were performed at 23 °C and 50% RH. The presented data correspond to the average value of seven test specimens.

To determine the water absorption, samples were dried in a desiccator at 0% RH for one week. Subsequently, they were placed in a beaker at 100% RH and allowed to absorb water until a constant weight was attained. Water uptake was calculated as:

[(*W_f_* − *W_i_*)/*W_i_*] × 100 (4)

where *W_i_* and *W_f_* are the initial and final (equilibrium) weight of the samples, respectively. Five replicates for each sample were measured, and the average value is reported.

The water vapour permeability (WVP) of the films was determined at 25 °C following the gravimetric method ASTM E96-95 standard [52] using Payne permeability cups of 3.5 cm diameter. Samples were equilibrated at 54% RH by using magnesium nitrate-6-hydrate. WVP was calculated according to the equation:

WVP = (Δ*m* × *l*)/(*A* × *t* × Δ*P*) (5)

where Δ*m* is the weight loss of each cup, *l* the thickness of the film, *A* the contact area, *t* the time and Δ*P* the partial pressure difference between inside and outside of the cup. For each sample, WVP measurements were replicated four times and the average value is reported.

Oxygen permeability (OP) was evaluated at 25 °C on films equilibrated at 54% RH by measuring the oxygen transference rate (OTR) with a gas permeability tester (Ox-Tran 1/50 System), following the ASTM D3985-05 standard [53]. OP was calculated following the expression:

OP = (OTR × *l*)/Δ*P* (6)

where *l* is the average film thickness and Δ*P* the difference between oxygen partial pressure across the film. Tests were performed in triplicate, and average values and standard errors are provided.

Overall migration tests were carried out in two liquid food simulants: ethanol 10% (*v*/*v*) (simulant A) and isooctane (alternative simulant to D2). Nanocomposite films were immersed in a beaker with 10 mL of ethanol and kept in a controlled chamber at 40 °C during 10 days, according to the Commission Regulation EU No 10/2011 [54]; analogously, samples were immersed in isooctane and maintained at 20 °C for 2 days, following the EN-1186-1 standard [55]. Subsequently, the films were removed, the simulants were evaporated and the residue was weighed using a Sartorious 6080 electronic balance (Sartorious, Göttingen, Germany) with readability of 0.001 mg in order to determine the overall migration. Experiments were performed in triplicate and values are reported as mean ± standard deviation.

The antibacterial activity of the composites was tested against two test organisms: Gram-positive *Staphylococcus aureus* (*S. aureus*, ATCC 12600) and Gram-negative *Escherichia coli* (*E. coli*, ATCC 25922). All the samples were sterilized in an autoclave prior to the tests, and then submerged in a 3-day-old nutrient broth of ~2.0 × 10^6^ colony forming units per ml (CFU/mL). After incubation at 37 °C for 24 h, the number of viable microorganism colonies was counted manually using a pen and a click-counter, and the results were expressed as mean CFU/sample. The survival ratio (SR) was calculated using the equation: SR = (*N*/*N_o_*) × 100, where *N_o_* and *N* are the mean number of bacteria on the pure PHB and the nanocomposites, respectively. The tests were conducted in triplicate and the average values are reported.

## 4. Conclusions

Novel PHB/ZnO biodegradable nanocomposites were successfully prepared via a simple solution casting method without the aid of coupling agents, and their morphology, thermal, mechanical, barrier, migration and antibacterial properties were evaluated. FT-IR spectra demonstrated the existence of strong hydrogen bonding interactions between the surface hydroxyl moieties of the nanoparticles and the carbonyl of the ester groups of PHB. SEM and TEM images revealed that ZnO nanoparticles were dispersed as single particles at low concentrations, while both as individual particles and as small clusters at 10.0 wt % loading. The nanoparticles significantly enhanced the heat resistance of the matrix, ascribed to their barrier effect that hindered the transport of decomposition products from the bulk of the composite to the gas phase. Moreover, they acted as nucleating agents, increasing the crystallization temperature and the level of crystallinity of PHB. DMA tests showed a remarkably stiffening effect upon addition of ZnO, particularly at temperatures below the glass transition. The stiffness, strength and toughness of the biopolymer were simultaneously enhanced at low nanoparticle loadings, due to the homogenous nanofiller dispersion and its strong interfacial adhesion with the matrix through hydrogen bonding interactions. The water uptake, water vapour and oxygen permeability of PHB decreased by up to 66%, 38% and 53%, respectively, at 5.0 wt % ZnO. The migration levels of PHB/ZnO composites in both non-polar and polar simulants dropped with increasing nanoparticle content, and were well below the limits required by the current normative for food packaging materials. The nanocomposites displayed significant antibacterial activity against human pathogenic bacteria, which was progressively improved upon increasing ZnO concentration, and the effect on *E. coli* was systematically stronger than on *S. aureus*. The optimum balance between mechanical, barrier, migration and antibacterial properties was obtained at a critical ZnO concentration of 5.0 wt %. These bionanocomposites with antimicrobial function are an interesting alternative to synthetic plastic packaging materials, and are highly useful to minimize the growth of contaminant microorganisms, extending the shelf-life of food and improving its quality. In particular, they are suitable for use in packaging and disposable applications such as beverage and food containers, disposable cutlery, cups and utensils, blister packages, overwrap and lamination films.
